# Machine learning for genomic and pedigree prediction in sugarcane

**DOI:** 10.1002/tpg2.20486

**Published:** 2024-06-26

**Authors:** Minoru Inamori, Tatsuro Kimura, Masaaki Mori, Yusuke Tarumoto, Taiichiro Hattori, Michiko Hayano, Makoto Umeda, Hiroyoshi Iwata

**Affiliations:** ^1^ Laboratory of Biometry and Bioinformatics, Department of Agricultural and Environmental Biology, Graduate School of Agricultural and Life Sciences The University of Tokyo Tokyo Japan; ^2^ Toyota Motor Corporation, New Business Planning Division, Agriculture & Biotechnology Business Department Toyota Japan; ^3^ Toyota Motor Corporation, Environment Affairs and Engineering Management Division, CN Advanced Engineering Development Center Tokyo Japan; ^4^ NARO Kyushu Okinawa Agricultural Research Center, Tanegashima Sugarcane Breeding Site Nishinoomote Japan; ^5^ NARO Kyushu Okinawa Agricultural Research Center, Itoman Resident Office Itoman Japan; ^6^ NARO Institute for Agro‐Environmental Science Tsukuba Japan

## Abstract

Sugarcane (*Saccharum* spp.) plays a crucial role in global sugar production; however, the efficiency of breeding programs has been hindered by its heterozygous polyploid genomes. Considering non‐additive genetic effects is essential in genome prediction (GP) models of crops with highly heterozygous polyploid genomes. This study incorporates non‐additive genetic effects and pedigree information using machine learning methods to track sugarcane breeding lines and enhance the prediction by assessing the degree of association between genotypes. This study measured the stalk biomass and sugar content of 297 clones from 87 families within a breeding population used in the Japanese sugarcane breeding program. Subsequently, we conducted analyses based on the marker genotypes of 33,149 single‐nucleotide polymorphisms. To validate the accuracy of GP in the population, we first predicted the prediction accuracy of the best linear unbiased prediction (BLUP) based on a genomic relationship matrix. Prediction accuracy was assessed using two different cross‐validation methods: repeated 10‐fold cross‐validation and leave‐one‐family‐out cross‐validation. The accuracy of GP of the first and second methods ranged from 0.36 to 0.74 and 0.15 to 0.63, respectively. Next, we compared the prediction accuracy of BLUP and two machine learning methods: random forests and simulation annealing ensemble (SAE), a newly developed machine learning method that explicitly models the interaction between variables. Both pedigree and genomic information were utilized as input in these methods. Through repeated 10‐fold cross‐validation, we found that the accuracy of the machine learning methods consistently surpassed that of BLUP in most cases. In leave‐one‐family‐out cross‐validation, SAE demonstrated the highest accuracy among the methods. These results underscore the effectiveness of GP in Japanese sugarcane breeding and highlight the significant potential of machine learning methods.

## Core Ideas

- A numerator relationship matrix can be used as input for machine learning models.
- A new method named simulated annealing ensemble (SAE) to handle interactions among markers was developed.
- Machine learning methods, random forest and SAE, often outperformed the prediction accuracy of best linear unbiased prediction (BLUP).

**Abbreviations**
BLUP: best linear unbiased prediction
GRAS‐Di: genotyping by random amplicon sequencing, direct
GS: genomic selection
KARC: Kyushu Okinawa Agricultural Research Center
MCMC: Markov chain Monte‐Carlo
NARO: National Agriculture and Food Research Organization
NGS: next generation sequencing
PCR: polymerase chain reaction
RF: random forest
SAE: simulation annealing ensemble
SD: stalk diameter
SL: stalk length
SN: stalk number
SNP: single nucleotide polymorphism
SSC.M: soluble solid content in the middle part
SSC.U: soluble solid content in the upper part

## 1 INTRODUCTION

Sugarcane (*Saccharum* spp.) is a major industrial crop cultivated in tropical and subtropical regions of the world. Sugarcane accounts for nearly 80% of the global sugar production (https://www.isosugar.org/sugarsector/sugar). In recent years, sugarcane has attracted attention as a sustainable source of bioenergy. Research has progressed from first‐generation ethanol production using sucrose to second‐generation ethanol production through the saccharification of cell wall sugars (Calderan‐Rodrigues et al., 2021; Kandel et al., 2018). Sugarcane biomass can also be used for the production of high‐value compounds, such as hydrogen, cellulose, lignin‐derived products, organic acids, and bioplastics (Klein et al., 2019). Despite the potential and importance of sugarcane, recent improvements in its productivity have been trivial compared with those in other major crops. The rate of improvement in genetic gains from sugarcane breeding programs has stagnated because of narrow genetic bases, poor synchronization and fertility of flowers, long breeding cycle, low heritability of key agronomic traits, and high complexity and large size of the sugarcane genome (Kandel et al., 2018; Yadav et al., 2020). Thus, the efficiency of sugarcane breeding must be improved to fully exploit its potential and realize novel applications, such as biomass and energy production.

Genomic selection (GS; Meuwissen et al., 2001) has recently garnered considerable attention as a method for improving the efficiency of plant and animal breeding (Hickey et al., 2017; R2D2 Consortium et al., 2021). In GS, the breeding value (or genotypic value) of a target trait is predicted based on genome‐wide marker polymorphisms, and individuals/lines are selected based on the predicted value. While phenotyping remains a vital step in establishing accurate predictive models, some stages can be bypassed in genomic‐assisted programs, potentially accelerating the breeding cycle without compromising the importance of phenotyping (Heffner et al., 2009; Jannink et al., 2010; Lorenz et al., 2011). The long breeding cycle and low heritability of major agronomic traits hinder its breeding efficiency. GS has advantages over conventional selection methods for improving traits with low or moderate heritability that are dominated by many genes; thus, the introduction of GS into sugarcane breeding programs is expected to significantly contribute to the genetic improvement of this crop (Mahadevaiah et al., 2021; Voss‐Fels et al., 2021; Yadav et al., 2020).

However, the highly heterozygous polyploid genomes of sugarcane remain a hindrance in the application of GS in breeding programs, rendering the application of high‐throughput genotyping difficult; thus, careful data analysis with special algorithms is imperative (Garcia et al., 2013). Even if genotyping is performed accurately, the mode of inheritance of a trait of interest may be complex. In addition to additive ones, nonadditive genetic factors may affect the trait owing to the dominance and/or epistatic effects (Yadav et al., 2020, 2021). Therefore, technologies and methods must be developed to address these issues when applying GS in sugarcane breeding programs. To date, several studies have assessed the potential of GS in sugarcane production. For instance, Gouy et al. (2013) generated genomic predictions of 10 traits using 167 genotypes from each of the two genetic resource panels representing the genetic diversity of sugarcane worldwide. This study used 1499 diversity arrays technology markers and achieved an accuracy of genomic prediction of 0.1–0.6. Deomano et al. (2020) constructed prediction models for two yield traits using three panels (467, 1146, and 738 genotypes) from breeding populations. Approximately 50,000 single‐nucleotide polymorphisms (SNPs) from the Affymetrix Axiom array were used as markers, and the prediction accuracy was 0.2–0.5. Furthermore, Hayes et al. (2021) constructed models to predict yield‐ and flowering‐related traits using 3984 genotypes using 26,000 markers from the Affymetrix Axiom array and achieved a prediction accuracy of 0.2–0.5. Yadav et al. (2021) constructed a prediction model for three yield traits based on 3006 genotypes using 26,000 markers from the Affymetrix Axiom array and obtained a prediction accuracy of 0.2–0.5. Islam et al. (2021) constructed models to predict the susceptibility of two major diseases based on 432 genotypes using 8825 coding region‐based SNPs and achieved a prediction accuracy of 0.1–0.5. Although most of the above results were obtained for different materials and traits, all studies highlighted the potential for genomic prediction. In addition, Aono et al. (2020) constructed models to classify brown rust‐resistant and brown rust‐susceptible groups based on 219 full‐sib genotypes using 14,540 SNPs; the classification accuracy reached 95%, with 131 SNPs selected using feature selection methods. Voss‐Fels et al. (2021) simulated GS breeding in sugarcane and examined its effectiveness by considering the economic value of genetic improvement and the cost of genotyping. The authors noted that introducing GS into sugarcane breeding produced a significant effect. Collectively, these reports suggest a high potential for genomic selection in sugarcane.

In sugarcane, which has a highly heterozygous polymorphic genome, nonadditive genetic effects, that is, dominant and epistatic effects, must be considered in genomic prediction models (Yadav et al., 2021). Yadav et al. (2021) and Islam et al. (2021) showed that the accuracy of models that considered nonadditive genetic effects, albeit only for some traits, was higher than that of models that considered additive effects alone. Islam et al. (2021) demonstrated that the use of machine learning methods is effective for modeling nonadditive genetic effects (Bayer et al., 2021). Typically, machine learning methods possess excellent potential to incorporate nonadditive effects and may be useful for genomic selection in sugarcane. However, only a few studies (e.g., Islam et al., 2021) have applied machine learning methods for genomic prediction in sugarcane. In the present study, we examined the effectiveness of a novel machine learning method and a conventional method (random forest; RF). The former method can model nonlinear effects by explicitly incorporating interactions among variables (markers), while the latter method models nonlinear effects by combining variables as nodes of regression trees in a cascaded fashion.

**Core Ideas**

- A numerator relationship matrix can be used as input for machine learning models.
- A new method named simulated annealing ensemble (SAE) to handle interactions among markers was developed.
- Machine learning methods, random forest and SAE, often outperformed the prediction accuracy of best linear unbiased prediction (BLUP).

Sugarcane offers certain advantages over other crops in terms of genomic selection. As sugarcane breeding rarely uses inbred lines derived from repeated selfing and backcrossing, which are generally used in autogamous crops, the pedigree of breeding lines can be easily traced based on the relationship between parents and offspring. By tracing pedigrees, the degree of relatedness between genotypes can be calculated. Such pedigree relationships have been described for various crops to provide additional information for predicting the breeding (or genotypic) values and improving the accuracy of GS (de los Campos et al., 2009; Howard et al., 2019; Robertsen et al., 2019). In sugarcane, Deomano et al. (2020) conducted genomic prediction with and without pedigree information and demonstrated that pedigree information can improve the accuracy of GS. Although pedigree information may be useful for improving the accuracy of GS, several innovations are necessary to use this information in a machine learning method. This is because in genomic prediction, pedigree information is typically used as a matrix representing the relationships among genotypes (i.e., numerator relationship matrix) and not in the form of ordinary multivariate data. A simple yet effective approach to incorporate pedigree data may be to consider the relationship between a genotype and the others, that is, to consider each column of the numerator relationship matrix as a variable and use it as an input for the machine learning model. In other words, for a single genotype (clone/individual) for which the breeding (or genotypic) value is to be predicted, a vector with the same dimension as the number of genotypes in the training population is provided, which becomes the input vector for the machine learning model. Another potential method involves estimating the arrangement of genotypes in a multidimensional space through eigenvalue decomposition of the numerator relationship matrix and using the multidimensional coordinates as input for a machine learning model. These methods may be useful for combining pedigree information with genome‐wide marker genotypes in machine learning‐based genomic prediction model; however, their potential has not been evaluated to date.

Based on the perspectives described above, we examined the following attributes using a breeding population from the third round of selection in a Japanese breeding program as the experimental material: this (1) validates the prediction accuracy of best linear unbiased prediction (BLUP) based on genomic and pedigree information of the population, (2) investigates the use of pedigree information in machine learning models, and (3) proposes a novel machine learning method that can explicitly handle the interaction between genome‐wide markers and pedigree‐based genetic backgrounds. If the prediction accuracy of the machine learning methods surpasses that of BLUP, machine learning can be widely employed for prediction, including the utilization of a pedigree relationship matrix. To elucidate this, we conducted a comparative analysis of accuracy among different methods and inputs (genome and pedigree) using two distinct types of cross‐validation: repeated 10‐fold cross‐validation and leave‐one‐family‐out cross‐validation. Finally, we summarize the results of these studies and discuss the potential of genomic selection in future sugarcane breeding.

## 2 MATERIALS AND METHODS

### 2.1 Phenotype and marker genotype data

#### 2.1.1 Phenotypic data from a sugarcane breeding population

The present study analyzed phenotypic and marker genotype data collected from clones tested in the third round selection of the KARC/NARO sugarcane breeding program. Field trials of the lines were conducted at the Tanegashima Sugarcane Breeding Site of KARC/NARO (30°43′ N, 131°04′ E, altitude 45 m) from March 2018 to January 2019 (trial 1, T1) and from April 2019 to February 2020 (trial 2, T2). In each of T1 and T2, ∼150 third‐round selection clones and control cultivars (NCo310, NiF3, NiF8, NiTn18, and Kurokaido) were evaluated. As the breeding lines change every year, the third‐round selection clones in T1 and T2 were designated as K15 and K16, respectively, indicating groups that started selection at KARC/NARO in 2015 and 2016, respectively. Third‐round selection clones were chosen based on the Brix level and perspective growth rate during first‐ and second‐round selections. We utilized the third‐round selection clones because their population size is suitable for implementing GS, considering cost and a relatively large number of breeding combinations remained viable. The third‐round selection was performed in two fields with different soil types: “Kuroboku” and “Akahoya.” Kuroboku is an andsol found on Tanegashima Island and is characterized as volcanic ash soil. Akahoya also consists of volcanic ash but contains lower levels of K_2_O and organic matter than Kuroboku and is deposited beneath it. In both T1 and T2, ∼20 buds of each clone and cultivar were planted in a plot of 2.5 m × 1.1 m (73,000 plants ha^−1^) with one plot for each soil type.

In T1, basal fertilization and planting were performed on March 30, 2018. Fertilizer was applied twice: in mid‐June and mid‐July. In T2, base fertilization and planting were performed on April 1, 2019. Additional fertilization was applied on May 30 and June 10. In both trials, phenotype data related to sugarcane yield were collected during the harvest (late January to early February) period. The stalk number (SN, m^−2^) was counted for each plot. The stalk length (SL, cm) and stalk diameter (SD, mm) were measured in five medium‐sized stalks in each plot. The soluble solid content, measured as Brix level, was measured at the middle (SSC.M) and upper (SSC.U) parts of a stalk (%) for three medium‐sized stalks in each plot using a handheld refractometer (Master; ATAGO).

#### 2.1.2 Marker genotype data of the sugarcane breeding population

Sugarcane leaf samples (80–100 mg) were taken from the phenotyped plants and used for DNA extraction. The sampled leaf tissues were frozen in liquid nitrogen and placed in 2‐mL microcentrifuge tubes with three tungsten carbide beads (3 mm in diameter). The tissues were then crushed using a TissueLyser (QIAGEN) two times for 30 s at 27 vibrations per second. DNA was extracted from the obtained tissue powder using DNeasy Plant Mini Kit (QIAGEN).

Genotyping was performed using Genotyping by Random Amplicon Sequencing, Direct (GRAS‐Di), which was developed by Toyota Motor Corporation. GRAS‐Di is a next‐generation sequencing (NGS)‐based genotyping method that has recently become utilized in studies of various plant species (e.g., Miki et al., 2020; Moriya et al., 2021; Nakata et al., 2021; Nishiyama et al., 2023; Shirasawa et al., 2023; Yanar et al., 2023). Details of the method are provided in the patent (Enoki & Takeuchi, 2019) and the paper (Hosoya et al., 2019). This technology can obtain many SNPs evenly throughout the genome with high reproducibility and can also be applied to polyploid plants (Enoki, 2019; Umeda et al., 2021). Libraries for NGS were prepared using two rounds of PCR with template DNA from each sugarcane strain. The first PCR was performed using a primer mix of 12 random primers comprising 13‐mer sequences with 15 ng µL^−1^ of template DNA. The second PCR was performed in a 50‐µL reaction mixture containing 1.5 µL of the first PCR product from each strain and primers for the index sequence. The second PCR products were mixed at an equal volume in a tube, of which 50 µL was purified using the MinElute PCR Purification Kit (QIAGEN) and used as a sequence library. The sequence library was analyzed for each amplicon sequence using a next‐generation sequencer (HiSeq 4000; Illumina) under paired‐end conditions, with a read length of 100 bases. SNPs were genotyped using the GRAS‐Di software (Toyota Motor Corporation), which is currently commercially available. The software utilized NGS‐derived sequences and evaluated marker quality based on empirical criteria such as genotypic reproducibility, determined by the number of reads between samples and genotypic consistency (presence or absence of reads), using trial GRAS‐Di data for crop species. Markers were categorized into four ranks based on their reproducibility: A (reproducibility ≥ 99.99%), B (99.98% ≤ reproducibility < 99.99%), C (99.90% ≤ reproducibility < 99.98%), and D (99.80% ≤ reproducibility < 99.90%). SNP markers with a quality ranking of D or higher were selected using the software, and redundant markers showing identical genotypes were integrated. The process yielded a total of 33,149 markers. The genotypes of these markers were scored as 1 and 0 to denote the presence and absence of the allele, respectively.

### 2.2 Data analysis

#### 2.2.1 Genetic relationships among clones

Genetic relationships among clones were calculated using a pedigree‐based additive relationship matrix (**P**) and a genome‐wide marker‐based additive relationship matrix (**G**). In this study, we analyzed 297 clones from the sugarcane breeding population. These clones were distributed across 87 different families, with family sizes ranging from 1 to 21 clones. Eight families contained more than 10 clones, while 43 families contained only one clone. We calculated matrix **P** based on the sugarcane pedigree database (Tarumoto et al., 2016). The calculation of matrix **P** requires the pedigree information of clones in the breeding populations in the third round of selection as well as their ancestors. Relying solely on the clones within the breeding population could lead to inaccurate estimations of their relatedness, as the coefficient of inbreeding for founders (clones closest to the ancestors of the population) included in the data was assumed to be 0. The accuracy of the estimations was improved by including the ancestors of the clones targeted in this study, to account for any pre‐existing inbreeding or relatedness. In the calculation of matrix **P**, the coefficient of inbreeding of 0 is assumed for founders included in the data. We should include the ancestors of the clones in this study to take into account any preexisting inbreeding or relatedness. We calculated matrix **P** with 3892 clones, including 3595 ancestral clones and the 297 clones in the breeding population. Sugarcane exhibits high ploidy, but the level of ploidy varies from chromosome to chromosome, making it difficult to calculate matrix **P** given the level of ploidy. Therefore, we considered the clones to be diploid in the calculation of matrix **P**. We used the Amatrix function of R *AGHmatrix* (Amadeu et al., 2016) for calculations. The “ploidy” option of the Amatrix function was set to 2 such that the genome was considered diploid.

Matrix G was calculated as $G=\frac{{\mathrm{XX}}^{\prime }}{M}$, where X is the standardized genotype matrix (see below) and M is the number of SNPs (i.e., number of columns in matrix X). The elements of X in the ith column are the genotypic scores of marker i, which were standardized to have zero mean and unit variance, that is, $\sum_{j}^{N}\;x_{ij}=\;0$ and $\sum_{j}^{N}x_{ij}^{2}/N=1$ over N clones.

To summarize genetic relationships among the clones, we applied principal component analyses based on matrices **P** and **G**. With the eigenvalue decomposition of matrix **G**, we can perform principal component analysis based on X. Matrix **G** can be obtained by eigenvalue decomposition as $G\;=\;U_{G}\Lambda_{G}{U_{G}}^{\prime }$, where $U_{G}$ is a matrix of the eigenvectors (ith column is eigenvector ${u_{G}}_{i}$), and $\Lambda_{G}$ is a diagonal matrix with eigenvalues as diagonal elements (ith diagonal element is eigenvalue ${\lambda_{G}}_{i}$). The ith principal component score obtained from the principal component analysis of X can be expressed as $\sqrt{M{\lambda_{G}}_{i}}\;{u_{G}}_{i}$. Although matrix **P** does not have a corresponding matrix like **X** for matrix **G**, principal component analysis can be performed based on the eigenvalue decomposition of matrix **P**. The eigenvalue decomposition was performed as $P\;=\;U_{P}\Lambda_{P}{U_{P}}^{\prime }$, and the principal component scores were calculated as $\sqrt{{\lambda_{P}}_{i}}\;{u_{P}}_{i}$, where ${u_{P}}_{i}$ is the ith column of $U_{P}$ and ${\lambda_{P}}_{i}$ is the ith diagonal element of $\Lambda_{P}$.

#### 2.2.2 Estimation of genotypic values and heritability

The genotypic values of 297 lines were estimated using a mixed model. We estimated average genotypic values over two soil types (Kuroboku and Akahoya) based on the following fixed effect model:

(1) $$\;y_{ijkl}=m+g_{i}+t_{j}+s_{k}+e_{ijkl},$$

where $y_{ijkl}$ is the phenotypic value of observation l (l=1 for stalk number, l=1,2,3 for soluble solid content, and l=1,2,…,5 for the other traits) of genotype i(i=1,2,…,297) in yearly trial j(j=1,2) in soil type s (*s* = 1, 2), m is the overall mean, $g_{i}$ is the effect of genotype i, $t_{j}$ is the effect of yearly trial j, $s_{k}$ is the effect of soil type k, and $e_{ijk}$ is the error in observation l of genotype i in soil type k in trial j. All effects were treated as fixed effect. The soil‐type average genotypic value of genotype i, $\eta_{i}$, was estimated as ${\hat{\eta }}_{i}=\;\hat{m}\;+\;\hat{g_{i}}$, where $\hat{m}$ and ${\hat{g}}_{i}$ are the estimates of m and $g_{i}$, respectively.

Heritability was estimated based on the following mixed model:

(2) $$\;y_{ijkl}=m+g_{i}+t_{j}+s_{k}+gs_{ik}+e_{ijkl},$$

where $g_{i}$ is the random effect of genotype i, $gs_{ik}$ is the random effect of the interaction between genotype i and soil type k, and the definitions of the other symbols are the same as in the fixed effect model described above. After fitting the model, we obtained variance estimates for $g_{i}$, $gs_{ik}$, and $e_{ijk}$, that is, ${\hat{\sigma }}_{g}$, ${\hat{\sigma }}_{gs}$, and ${\hat{\sigma }}_{e}$, respectively. The heritability of traits other than stalk number was estimated as follows:

(3) $$\;H^{2}=\frac{{\hat{\sigma }}_{g}}{{\hat{\sigma }}_{g}+{\hat{\sigma }}_{gs}/K+{\hat{\sigma }}_{e}/KL}\;,$$

where K is the number of soil types (K = 2) and L is the number of observations per plot (L=3 or 5). The heritability of stalk number was estimated without considering the interaction between genotypes and soil types. We calculated the heritability using an approach that is not pedigree or genomic based. In this calculation, we assumed that random genotypic effect was independent among lines, that is, $g\;∼\;N(0,\;I\sigma_{g}^{2})$, where $g\;=\;{\left(g_{1},\;\text{…},\;g_{K}\right)}^{T}$ and $\sigma_{g}^{2}$ are the variances of the genotypic effect. Furthermore, we calculated pedigree‐ and genomic‐based heritability. For pedigree‐based heritability, we assumed that the random genotypic effect had a variance structure proportional to matrix **P**, that is, $g\;∼\;N(0,\;P\sigma_{g}^{2})$. Similarly, for genomic‐based heritability, we assumed a variance structure proportional to matrix **G**, that is, $g\;∼\;N(0,\;G\sigma_{g}^{2})$. For estimating the variance of random effects in the mixed model, we used the lmm.aireml function of the R package *gaston* (Dandine‐Roulland & Perdry, 2018).

The bootstrap method was employed to assess the standard error of the heritability estimates. From the original dataset used for estimating heritability, an equal number of data points were randomly sampled with replacement to create a set of bootstrap samples. A total of 100 sets of bootstrap samples were generated, and for each set, heritability was estimated following the same procedure as with the original data. The standard deviation of these estimates was then calculated across the 100 sets of bootstrap samples.

### 2.3 Prediction methods and models

#### 2.3.1 G and P BLUP

Models for predicting the genotypic values $\eta_{i}$ were constructed using BLUP and two machine learning methods: RF and simulation annealing ensemble (SAE). The BLUP model was set as follows:

(4) η=μ+α+β+γ+ε,

where η is the vector of genotypic values whose ith element is $\eta_{i}$ or $\eta_{ik}$; μ is the vector whose elements are the overall mean; α is the vector of genetic effects accounted by genomic‐based relationships, that is, $\alpha \;∼\;N(0,\;G\sigma_{G}^{2})$, where $\sigma_{G}^{2}$ is the variance of the genomic‐based effect; β is the vector of genetic effects accounted by pedigree‐based relationships, that is, $\beta \;∼\;N(0,\;P\sigma_{P}^{2})$, where $\sigma_{P}^{2}$ is the variance of the pedigree‐based effect; γ is the vector of genetic effects accounted by the interaction between genomic‐ and pedigree‐based relationships, that is, $\gamma \;∼\;N(0,\;G⊙P\sigma_{\mathrm{GP}}^{2})$, where $\sigma_{\mathrm{GP}}^{2}$ is the variance of the interaction effects and ⊙ is the element‐wise product (Hadamard product) of matrices; and ε is the vector of residuals assuming $\varepsilon \;∼\;N(0,\;I\sigma_{E}^{2})$, where $\sigma_{E}^{2}$ is the residual variance. This model incorporates the interaction terms between genomic and pedigree relationships and was first proposed by Howard et al. (2019). It is hereafter referred to as the G × P‐BLUP (BLUP.G × P) model. In addition, we applied the following three reduced models of G × P‐BLUP:

G‐BLUP (BLUP.G):

(5) η=μ+α+ε.

P‐BLUP (BLUP.P):

(6) η=μ+β+ε.

GP‐BLUP (BLUP.GP):

(7) η=μ+α+β+ε.

To estimate model parameters and calculate BLUP values, we used the R package *BGLR* (Pérez & de los Campos, 2014). Hyperparameters for the estimation and calculation were set to the default settings of the BGLR function. The number of Markov chain Monte Carlo (MCMC) iterations was set to 5,000 for the burn‐in period and 15,000 for the total period.

#### 2.3.2 Random forest

RF (Breiman, 2001) is an ensemble algorithm based on randomized regression trees. In RF, each regression tree is constructed using randomly selected explanatory variables based on randomly selected samples with replacement (i.e., bootstrap samples) from the training dataset. The final prediction is computed by averaging the predictions of all regression trees in the forest.

In the present study, we constructed an RF regression model using a relationship matrix computed from genome‐wide marker or pedigree data (**G** or **P**) as the input. Specifically, we considered one column of matrix **G** or **P** as an explanatory variable and input it into the RF model that predicted the genotype value of a trait. This model predicted the genotypic value based on the genetic relationships between the target (i.e., clones whose genotypic values were to be predicted) and other (i.e., clones in the training dataset) lines. For comparison, we also constructed an RF model using principal component scores obtained from the eigenvalue decomposition of matrix **G** or **P**. Principal component scores are described in the previous section. The principal component scores computed from matrix **G** or **P** were used as input for the RF model. For genome‐wide genotype marker data, we also assigned the marker genotype score X as an input to the RF; this is a usual method of using RF for genomic prediction. The RF models are referred as follows: RF using matrix G directly is RFM, RF using principal component scores based on eigenvalue decomposition of matrix G is RFP, and RF using marker scores is RFX. To estimate the parameters of the RF models, we used the ranger function of the R package *ranger* (Wright & Ziegler, 2017). The hyperparameters used in the RF modeling were set to the default values of the ranger function.

#### 2.3.3 Simulation annealing ensemble

In modeling epistasis, a possible approach is to explicitly model the interactions between markers. However, when the number of markers is high, such models must consider epistasis from a massive number of marker combinations, even if they are limited to second‐order additive–additive epistasis. For instance, in the present study, ∼30,000 DNA markers were used, and the number of combinations of these markers was ∼4.5 billion. From such many candidate variables, we developed a model to narrow down and select useful combinations for prediction and build a prediction model based on them.

In this method, we must first determine the number of variables that represent the main effect and the number of variables that represent the interaction. In the present study, the number of each variable group (main effect or interaction) was set to 10. Subsequently, a simulated annealing algorithm was applied to select important variables.

The state must be defined to apply the simulated annealing method. The state is defined as the current selected variables. Note that there are two types of interaction. For instance, if the variables are x and y, there are two types of interactions: xy, which is the product of the variables, and x+y−xy, which includes the main effect.

The energy of a state must also be defined. To reduce the normalized mean squared error of multi‐linear regressions with the 20 selected variables, we used the normalized mean squared error as energy. Specifically, the energy $E=\frac{10^{6}{∥p-\eta ∥}^{2}}{{\eta_{w}}^{2}n}$, where η is a vector of genotypic values, p is a vector of predicted genotypic values, n is the number of elements in the vector η and $\eta_{w}$ is a scalar value representing the range (difference between maximum and minimum) of the elements of η, such that, $\;\eta_{w}=\;\max\left(\eta \right)-\min\left(\eta \right)$.

State transitions must also be defined. We randomly selected a variable to be removed from the selected variables and another variable from the unselected variables to be the substitute of the removed variable. When the removed variable represented the main effect, the substitute variable was selected to represent the main effect. When the removed variable represented the interaction effect, the substitute variable was selected to represent the interaction effect.

We repeated the cycle involving the following steps.

Step 1. We randomly selected 20 variables. Of the 20 variables, 10 represented the main effect, and 10 represented the interaction effect.
Step 2. We determined the initial temperature such that the probability of state transition was 1/2 when the energy of the next state was higher than that of the initial state. Specifically, we randomly obtained 1000 energy differences between the initial and next states and obtained the median of energy differences from trials on a set of positive (increasing) energy differences. Here, the median is represented by dE. The probability of the state transition is $\exp(-\frac{dE}{T})$. Then $T_{\mathrm{init}}=\frac{dE}{\log2}\;$.
Step 3. We implemented a temperature reduction scheme such that the temperature decreased proportionally to difference from 0° and reached 1° at the 5000th step, following the formula:
(8) $$T=T_{\mathrm{init}}\;\exp\left(-\frac{\log T_{\mathrm{init}}}{t_{\max}}t\right),$$

where the time t progressed incrementally from 0 to $t_{\max}=\;5000$.

Step 3a. The process concluded when the set of variables remained unchanged for 500 consecutive steps. This termination criterion ensured that the algorithm ceased when the variables did not change over this duration.
Step 4. Subsequently, we identified the set of variables with the minimum energy.

These steps of simulated annealing were iterated 1000 times.

Predictions were generated through multiple regression on each variable set obtained using simulated annealing. The predictions were then weighted and averaged. Here, weighting can be performed using any method. In the present study, the coefficient of determination of each multiple regression was used. The advantage of this weighting method is that reliable results can be obtained without adjusting hyperparameters. Let $p_{i}$ be the result of multiple regression on each set of variables; then, the prediction value p is calculated as follows:

(9) $$p\;=\frac{p_{i}r_{i}^{2}}{\sum_{i}r_{i}^{2}}.$$

In SAE, for marker genotypes, the columns of the marker genotype score matrix **X** were used as explanatory variables. For pedigree information, the columns of the **P** matrix were used as explanatory variables, as in RF. When considering both marker genotypes and pedigree information, both the columns of the **X** matrix and the columns of the **P** matrix were used as explanatory variables.

#### 2.3.4 Validation of prediction accuracy

To evaluate the prediction accuracy of each method, cross‐validation was performed according to two schemes. The first cross‐validation scheme was based on random partitioning of all genotypes into 10 folds (10‐fold cross‐validation). In this scheme, nine folds were used to build the prediction model, and the remaining fold was predicted using the obtained model and used to evaluate the prediction accuracy. This process was repeated such that all folds could be subjected to prediction and evaluation. Since random splitting causes stochastic fluctuations in the values of prediction accuracy indices (see below), the same split was applied in all methods. In addition, 100 repetitions of random split were applied to deal with fluctuations.

The second scheme was based on “leave‐one‐family‐out” cross‐validation. The purpose of this cross‐validation scheme was to evaluate prediction accuracy for the genetic ability of offspring resulting from new mating combinations of parents. The 297 genotypes belonged to 87 families (Figure S1). We built a prediction model using data from 86 families, leaving one family out and evaluated prediction accuracy for genotypes included in the left‐out family using the obtained model. Since partitioning was performed deterministically and no stochastic fluctuation occurred in this scheme, this cross‐validation was performed only once.

Prediction accuracy was evaluated based on Pearson's product moment correlation coefficient between the predictions and BLUP of genotypic values. The predicted values calculated in one repetition of cross‐validation were aggregated for all genotypes, and the aggregated values were compared with the corresponding BLUP of the genotypic values. Thus, in the 10‐fold cross‐validation, 100 values of the correlation coefficient were calculated for the 100 repetitions, whereas one value was calculated in the leave‐one‐out cross‐validation. In the 10‐fold cross‐validation, as described above, the same split was applied to all methods, ensuring consistent comparison of correlation coefficients for each repetition. Consequently, we computed the following two indices: “superiority” and “difference in accuracy.” For superiority, we compared the correlation coefficients between pairs of methods by assessing how often, out of 100 repetitions, one method exhibited a larger correlation coefficient than the other. We then divided this count by 100 to determine the superiority of each method. Regarding difference in accuracy, we computed the difference in correlation coefficients for all pairs of methods for each repetition and then calculated the average of these standardized differences over 100 repetitions.

## 3 RESULTS

### 3.1 Genetic relationships among individuals in the breeding population

**FIGURE 1:**
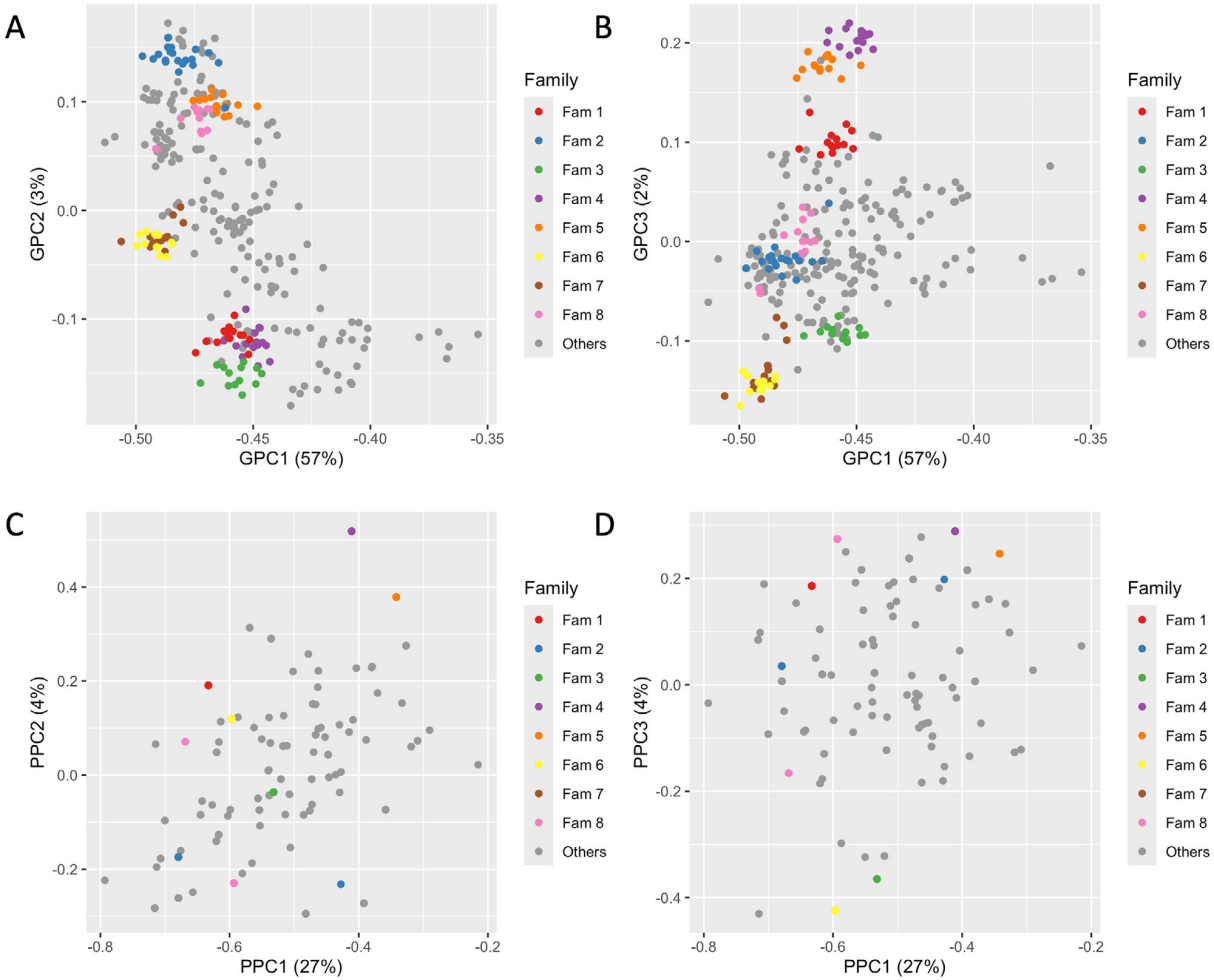
Scatter plot of principal component scores of genotypes. The first and second components (A) and the first and third components (B) based on the genomic relationship matrix (GPC1 and 2). The first and second components (C) and the first and the third components (D) based on the pedigree relationship matrix (PPC1 and 2). Eight families (Fam 1–Fam 8) containing more than 10 genotypes are colored.

We calculated a genomic relationship matrix based on polymorphisms of genome‐wide markers and a pedigree‐based relationship matrix based on parent–offspring relationship information of 297 genotypes used in the analysis. Figure 1 shows a scatter plot of principal component scores based on the genomic‐ and pedigree‐based relationship matrices; eight families containing more than 10 clones are colored. In principal component analysis based on the pedigree‐based relationship matrix, genotypes belonging to each family are represented as a single point. The proportion of variation explained by the first five principal components was 63.8% in the genomic relationship matrix and 40.9% in the pedigree‐based relationship matrix. Consequently, there was an association observed between the two sets of principal components derived from genomic‐ and pedigree‐based relationship matrices. Moderate to strong correlations (0.4<|r|≤0.7 and |r|>0.7, respectively) were noted across various combinations (see Figure S1).

### 3.2 Heritability

**FIGURE 2:**
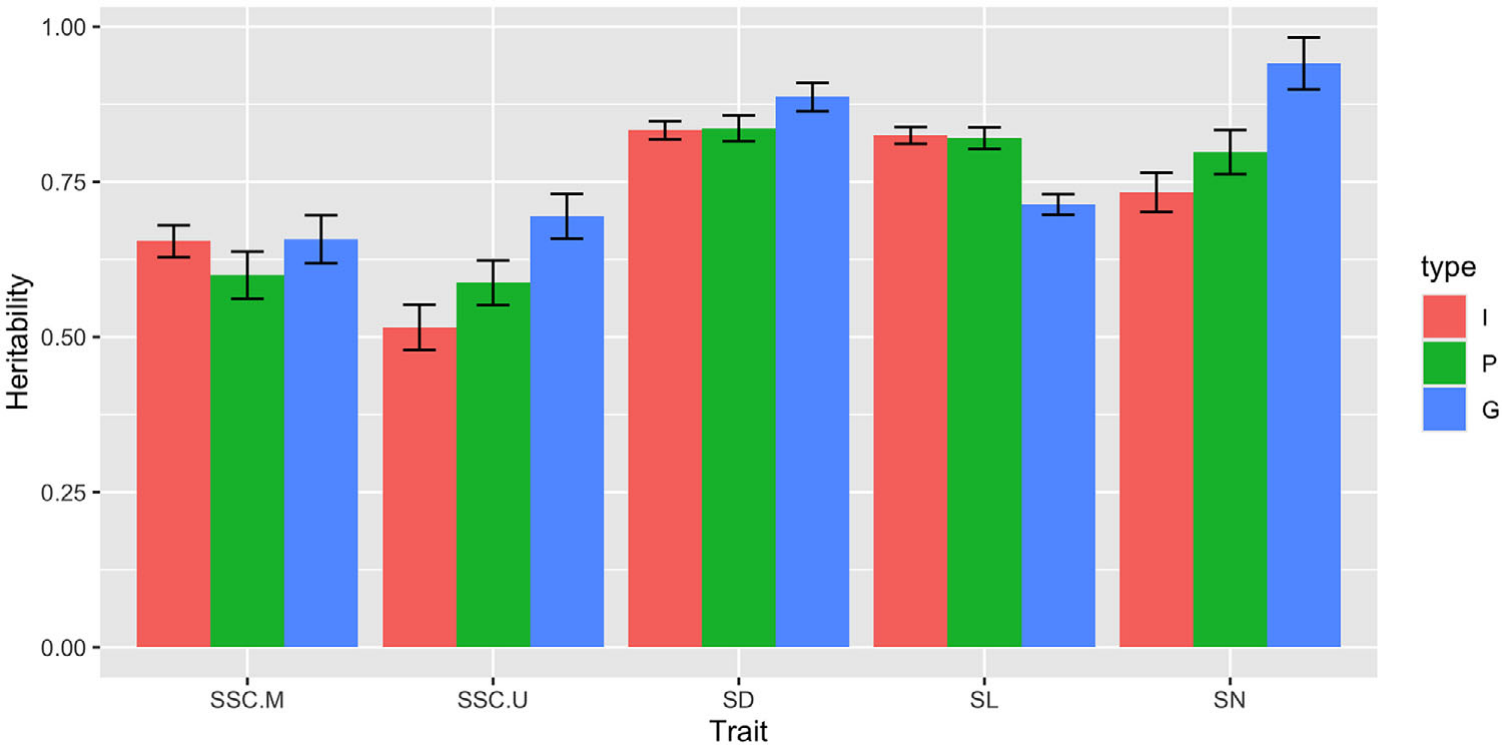
Estimates of the heritability of five target traits under three different assumptions of random genetic effects: (I) effects independently follow identical normal distribution; (P) heritability assuming the among‐line covariance structure according to matrix **P**; and (G) heritability assuming the among‐line covariance structure according to matrix **G**. This heritability is called “genomic heritability.” The error bars represent the standard deviation of the estimated heritability. The standard deviation was estimated over the 100 sets of bootstrap samples. SD, stalk diameter; SL, stalk length; SN, stalk number; SSC.M, soluble solid content in the middle part; SSC.U, soluble solid content in the upper part.

Similar estimates of heritability were obtained under the assumptions that genotype effects are independent and that the effects have a covariance structure that follows the pedigree‐based or genomic relationship matrix (Figure 2). Heritability estimated based on the genomic relationship matrix showed the highest values for SSC.U, SD, and SN and the lowest value for SL. The heritability of stalk‐related traits (SD, SL, and SN) exceeded 0.7 and was estimated to be approximately 0.8 in most cases. In contrast, the heritability of SSC was lower than 0.7 for SSC.M and SSC.U.

### 3.3 Accuracy of genomic predictions with BLUP based on matrix G

**FIGURE 3:**
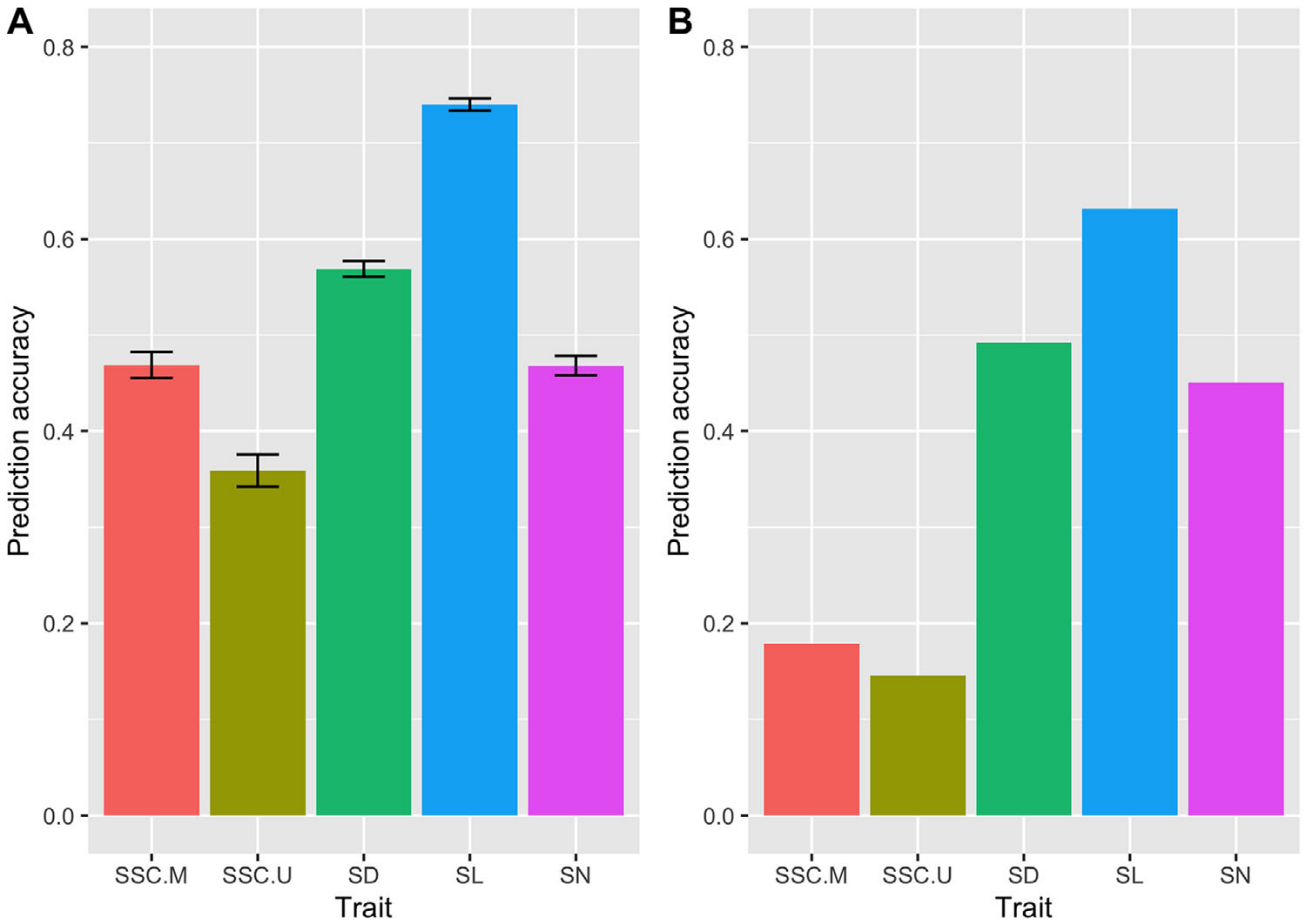
Prediction accuracy of best linear unbiased prediction (BLUP) based on matrix **G** (BLUP.G). (A) Prediction accuracy in 10‐fold cross‐validation. The error bars represent the standard deviation of the prediction accuracy over 100 repetitions of the cross‐validation. (B) Prediction accuracy in leave‐one‐family‐out cross‐validation. The prediction accuracy was calculated as Pearson's product moment correlation coefficient between the estimated and predicted genotypic values. SD, stalk diameter; SL, stalk length; SN, stalk number; SSC.M, soluble solid content in the middle part; SSC.U, soluble solid content in the upper part.

Figure 3 depicts the prediction accuracy of BLUP based on matrix G (BLUP.G) for each trait. Across all traits, we observed lower accuracy in the leave‐one‐family‐out cross‐validation (Figure 3B) compared to the 10‐fold cross‐validation (Figure 3A). Notably, SSC.M and SSC.U exhibited a 60% reduction in accuracy in the leave‐one‐family‐out cross‐validation relative to the 10‐fold cross‐validation. Among the five traits, SL demonstrated the highest prediction accuracy in both cross‐validations, scoring 0.74 in the 10‐fold cross‐validation and 0.63 in the leave‐family‐out cross‐validation. SD exhibited the second highest prediction accuracy at 0.57 in the 10‐fold cross‐validation and 0.49 in the leave‐one‐family‐out cross‐validation. SSC.U exhibited the lowest accuracy at 0.36 in the 10‐fold cross‐validation and 0.15 in the leave‐one‐family‐out cross‐validation. Moreover, we conducted 100 repetitions of 10‐fold cross‐validation, revealing that the standard deviations were exceedingly small compared to the mean differences between traits (Figure 3A).

### 3.4 Comparison of prediction accuracy among models

**FIGURE 4:**
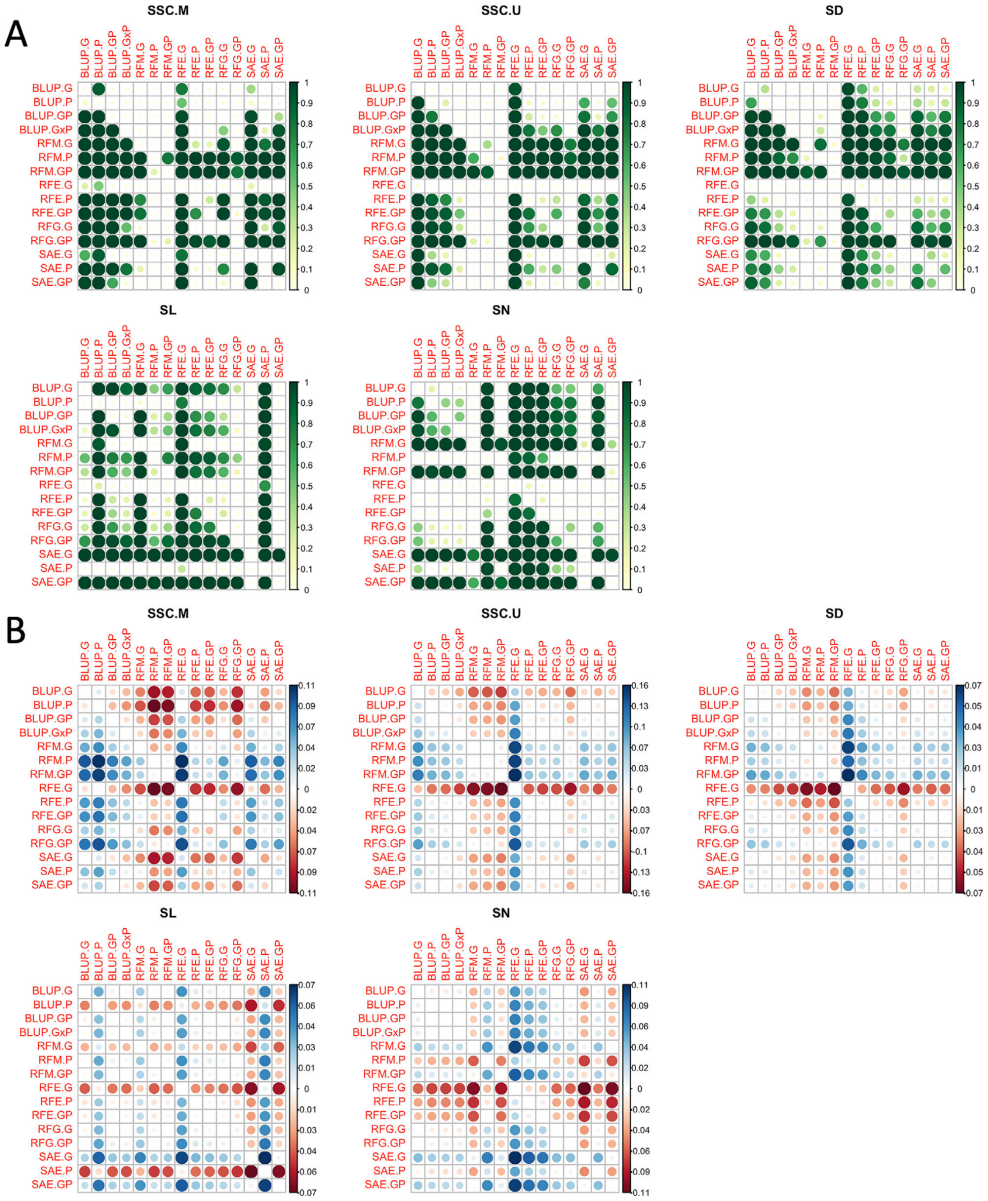
Accuracy comparisons between models in the 100 repetitions of 10‐fold cross‐validation. (A) The “superiority” index representing the proportion of row‐side models that outperformed column‐side models over 100 repetitions and (B) the “accuracy difference” index representing the average difference in accuracy between the row‐side model and the accuracy of the column‐side model. Positive values (blue) indicate that the row‐side models exhibit higher accuracy compared to the column‐side models. Models G, P, and GP denote models using matrix G, matrix P, and both matrices G and P, respectively. Model GxP represents the BLUP using an interaction matrix between G and P in addition to matrices G and P. The method names are as follows: BLUP (best linear unbiased prediction), RF using matrix (or matrices) as direct input RFM, RF using principal component scores based on eigenvalue decomposition of matrix (or matrices) as input (RFP), RF using marker scores as input (RFX), and SAE (a novel machine learning method proposed in this study). SAE, simulation annealing ensemble; SL, stalk length; SN, stalk number.

Figure 4 illustrates the comparison of prediction accuracy between models based on 10‐fold cross‐validation with 100 iterations. We compared the accuracies among the four models in BLUP. For SSC.M, SSC.U, and SD, BLUP.GxP demonstrated higher accuracies than other BLUP models, with the superiority values to the other models reaching 1. Similarly, for SN, BLUP.GxP exhibited the highest accuracy among BLUP models, although the difference in accuracy between BLUP.GxP and BLUP.GP was marginal (52 out of 100 times higher accuracy). Conversely, in SL, BLUP.G demonstrated higher accuracy than other BLUP models, with the superiority values to the other models surpassing 0.85. These findings underscore the significance of incorporating the P matrix in the BLUP method, albeit necessitating its simultaneous use with G matrix information.

Comparing the accuracy of the machine learning models (RFM, RFE, RFX, and SAE), it is notable that RFM and SAE exhibited relatively high accuracy. BLUP was not the most accurate for any trait.

For the trait SSC.M, RFM.P was the most accurate among the models, outperforming RFM.GP, the next most accurate, in 85 out of 100 instances. For traits U and SD, RFM.GP was the most accurate among the models, surpassing the next most accurate model in more than 84 out of 100 instances. For traits SL and SN, SAE.G demonstrated the highest accuracy among all models, outperforming SAE.GP, the next most accurate model, in all 100 instances for SL, and outperforming RFM.G, the next most accurate model, in 82 out of 100 instances for SN.

RFE and RFX generally displayed lower accuracy than RFM. Notably, when only genomic data were utilized, RFM.G, which utilizes the columns of the genomic relationship matrix as variables, was more accurate than RFX.G, which employs typical genomic data, except for SL. Additionally, RFE.G, which employs principal components of the genomic relationship matrix as variables, was the least accurate among models across all traits.

**FIGURE 5:**
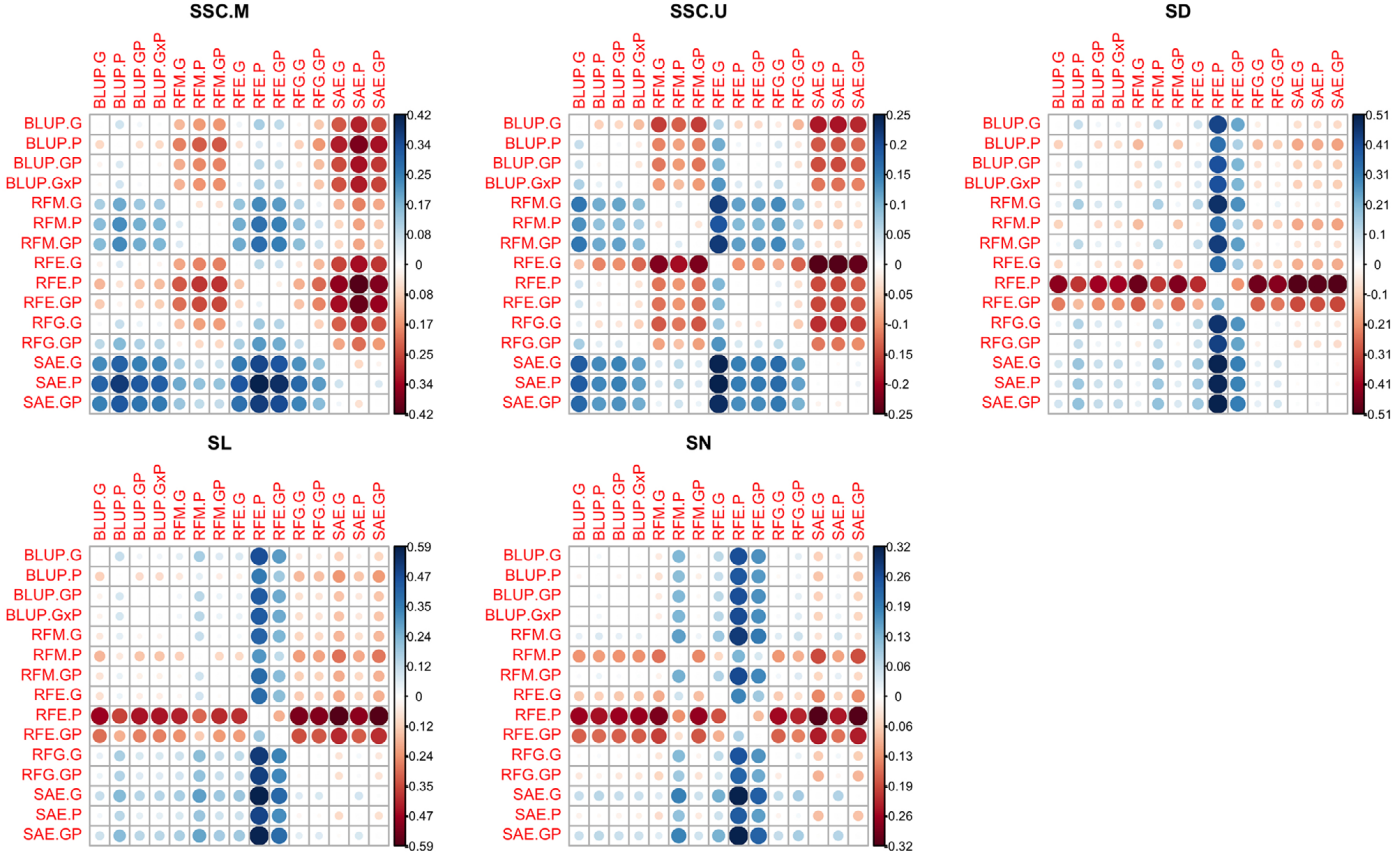
“Accuracy difference” index representing the difference in accuracy between the row‐side and column‐side models. Positive values (blue) indicate that the row‐side models exhibit higher accuracy compared to the column‐side models. Models G, P, and GP represent models using matrix **G**, matrix **P**, and both matrices **G** and **P**, respectively. Model GxP represents the BLUP using interaction matrix between G and P in addition to matrices G and P. Method names are as follows: BLUP, best linear unbiased prediction, RF using matrix (or matrices) as direct input (RFM), RF using principal component scores based on eigenvalue decomposition of matrix (or matrices) as input (RFP), RF using marker scores as input (RFX), and SAE (a novel machine learning method proposed in this study). SAE, simulation annealing ensemble; SL, stalk length; SN, stalk number.

Figure 5 depicts the differences in accuracy between models. A consistent pattern of superiority or inferiority among models across traits can be observed, with similar trends for SSC.M and SSC.U, as well as for SD, SL, and SN. For traits SSC.M and SSC.U, RFM and SAE exhibited high accuracy, particularly SAE. Within RFM and SAE, there were no significant differences in accuracy among G, P, and GP models. For SD, SL, and SN, there were generally no notable differences in accuracy between methods except for instances of notably low accuracy observed for RFE.P, RFE.GP, SN, and RFM.P. However, SAE demonstrated higher accuracy in most cases, albeit with minor differences. For all traits, none of the four BLUP models demonstrated the highest accuracy.

## 4 DISCUSSION

### 4.1 Genomic prediction accuracy

Genomic prediction using G‐BLUP (BLUP.G) exhibited a prediction accuracy of approximately 0.15–0.74 for stalk biomass and sugar content (Figure 3). However, notable discrepancies were noted based on the cross‐validation method employed. Specifically, the accuracy ranged from 0.36 to 0.74 with 10‐fold cross‐validation, whereas it was lower, ranging from 0.15 to 0.63 with leave‐one‐family‐out cross‐validation. The difference in accuracy between the two cross‐validation methods can be attributed to the loss of information on full‐sibs in the training data when employing leave‐one‐family‐out cross‐validation. When excluding each of the 10 families containing more than 10 clones, one full‐sib clone was left in the training dataset, and the cross‐validation approach resulted in improved accuracy (see Figure S2). Note that in Japanese sugarcane breeding, 10‐fold cross‐validation serves as a validation scheme that reflects the actual breeding program. This is due to the frequent use of identical parental combinations and the inclusion of full‐sib data in the training dataset in many instances.

Moreover, the prediction accuracy varied among traits, as illustrated in Figure 3. Notably, the prediction accuracy was higher for the stalk biomass, exhibiting higher precision for SL and comparatively lower precision for SN. Conversely, predictive accuracy for sugar content trailed that of the stalk biomass, particularly when applying leave‐one‐family‐out cross‐validation. Estimates of heritability presented in Figure 2 indicated a higher heritability for the stalk biomass than for sugar content. This suggests that the superior accuracy of genomic predictions aligns with the level of heritability for each trait.

In addition, a comparison of accuracy between methods and models through cross‐validation provided the following insights. First, the inclusion of pedigree information for prediction suggested the potential for accuracy enhancement. For instance, in 10‐fold cross‐validation, the P and GP models generally exhibited higher accuracy than the G model within the same method, with a few exceptions (Figure 4). Notably, for SSC.M, SSC.U, and SD, RFM demonstrated the highest accuracy among methods when incorporating pedigree information via RFM.P and RFM.GP. For SL and SN, SAE showed the highest accuracy among methods, although SAE.P showed lower accuracy than SAE.GP and SAE.G. A similar trend was observed even in leave‐one‐family‐out cross‐validation, although the pattern of superiority in SD for RFM differed, with RFM.G outperforming RFM.P and RFM.GP. These findings underscore that, notwithstanding exceptions based on trait–method combinations, leveraging pedigree information proves advantageous for populations with well‐established pedigree data, such as this sugarcane breeding population, due to its potential to enhance prediction accuracy.

The difference in prediction accuracy between the machine learning method and BLUP was evident from the results of repeated 10‐fold cross‐validation. Across all five traits, the machine learning method consistently outperformed BLUP in accuracy. Although the lack of repetition in leave‐one‐family‐out cross‐validation complicates a clear demonstration of the results, it can be inferred that the machine learning method is at least as accurate as BLUP. This implies that by incorporating pedigree information alongside genomic data, machine learning methods can achieve equal or superior accuracy than BLUP based on genomic and pedigree relationship matrices.

### 4.2 Comparison of genomic prediction accuracy with previous reports

The accuracy of genomic prediction varies depending on the test population. In the present study, we evaluated the accuracy of genomic predictions using Japanese breeding populations. Deomano et al. (2020), Hayes et al. (2021), and Yadav et al. (2021) used Australian breeding populations, while Islam et al. (2021) used an American breeding population. In these studies, the genetic variation was ∼0.1–0.5, similar to the prediction accuracy noted in the present study. Although prediction accuracy differs among traits with different genetic systems, the prediction accuracy for traits evaluated in the present study was similar to that for yield and disease resistance traits evaluated in previous studies.

Some previous studies did not observe significant differences among the modeling methods applied. For instance, in Gouy et al. (2013), the four methods used exhibited similar accuracies. Hayes et al. (2021) noted improved prediction accuracy with methods that used both pedigree and genomic information, although the difference was not significant. In contrast, Yadav et al. (2021) applied G‐BLUP that considered nonadditive effects (dominance effect and additive–additive epistasis) in the model and noted improved prediction accuracy for one of the three traits. Similarly, Islam et al. (2021) observed improved prediction accuracy with nonadditive machine learning‐based models. In this study, leave‐one‐family cross‐validation revealed that RFM exhibited higher prediction accuracy than additive G‐BLUP (BLUP.G in this study) for traits SSC.M and SSC.U, as well as SAE for all traits, suggesting potential contributions of non‐additive effects to these traits. Specifically, for SSC.M and SSC.U, RFM and SAE demonstrated correlation coefficients 0.1–0.3 higher than those of BLUP.G. However, for traits SD, SL, and SN, there was no significant enhancement in prediction accuracy observed with nonlinear machine learning methods. Nevertheless, it is noteworthy that in all traits, the nonlinear machine learning methods outperformed additive G‐BLUP, highlighting its superiority in prediction accuracy.

Notably, the prediction accuracy varied depending on the cross‐validation method. Hayes et al. (2021) and Yadav et al. (2021) used “forward prediction” rather than cross‐validation. In those studies, prediction accuracy was evaluated in a forward manner, that is, in the previous year (Hayes et al., 2021) or 2 years (Yadav et al., 2021) of available breeding data. Such prediction may be more difficult than cross‐validation of data from the same year because of the genotype × environment (year) effect (Hayes et al., 2021) and genetic shifts in the breeding populations over years (i.e., genetic differentiation among populations of different years). In the present study, we did not use forward prediction; however, since prediction accuracy in leave‐one‐family‐out cross‐validation was significantly reduced in our study, genetic shifts between years may reduce the accuracy of forward prediction.

### 4.3 Pedigree information used in machine learning models

RFs have been frequently used for genomic prediction and are accurate as models based on quantitative genetic methods (Blondel et al., 2015; Heslot et al., 2012; Ogutu et al., 2011; Onogi et al., 2015; Spindel et al., 2015). However, it is difficult to use information that specifies the covariance structure between phenotypes, such as a numerator relationship matrix, simultaneously with genomic information. In the present study, we showed that a numerator relationship matrix can be applied with an RF model using the corresponding column (i.e., column corresponding to the target genotype) of the matrix as the input vector. To the best of our knowledge, no such method has been proposed in RF for genomic prediction. Furthermore, a genomic relationship matrix could be constructed in the same way using the corresponding column as the input vector. The accuracy of this method (RFM) surpassed that of RFX, which directly utilized genomic data. When dealing with a vast number of markers, employing the corresponding columns of the genomic relationship matrix as input might prove more effective than utilizing genomic data directly. It is important to note that both the numerator relationship matrix and genomic relationship matrix can be represented as multidimensional data through spectral decomposition. However, the prediction accuracy of the method using variables after spectral decomposition (RFE) was inferior to that of RFM and RFX.

RFs are effective in the genomic prediction of disease resistance in sugarcane (Aono et al., 2020; Islam et al., 2021). In addition to genomic information, pedigree information can be used in such models. In addition, Rutkoski et al. (2012) demonstrated the effectiveness of RFs in which phenotypes of secondary traits were used as the input in addition to genomic data. According to Rutkoski et al. (2012), the usefulness of RFs lies in their flexible modeling capability. With the feasibility of using pedigree information for modeling, the possibilities of using RF may expand.

### 4.4 Efficacy of SAE over other methods

We proposed SAE, a method for variable selection that explicitly incorporates interactions. SAE demonstrated prediction accuracy comparable to or even superior to RF for some traits. Through 10‐fold cross‐validation, SAE emerged as the optimal method for SN and SD. Furthermore, leave‐one‐family‐out cross‐validation demonstrated SAE as the superior choice for all traits, displaying a smaller decrease in accuracy compared to other methods when transitioning from 10‐fold cross‐validation. These findings suggest that SAE is promising as an approach for capturing nonlinear relationships among markers. Moreover, akin to RF, SAE can also utilize the columns of the pedigree relationship matrix as variables. Notably, for certain traits such as SSC.M and SSC.U, the accuracy achieved with pedigree‐based SAE surpassed that attained with genome‐based SAE.

### 4.5 Potential of genomic prediction in sugarcane breeding

The polyploid genome and high heterozygosity of sugarcane has been a barrier to selective breeding. Genomic selection is expected to streamline sugarcane breeding if it enables more accurate predictions. This study demonstrated the effectiveness of genomic prediction and selection in sugarcane, as suggested in previous research (Deomano et al., 2020; Gouy et al., 2013; Hayes et al., 2021; Yadav et al., 2021). Pedigree information is conserved in sugarcane breeding. The utilization of such pedigree information has also been shown to enhance prediction accuracy. Previous studies represented relationships between clones/varieties using pedigree information as a pedigree‐based relationship matrix and further represented it as the covariance structure of variable effects in mixed models. In this study, by using columns of the pedigree‐based relationship matrix and/or the genomic relationship matrix as input variables for machine learning models, we demonstrated that prediction with accuracy equivalent to BLUP or even higher is feasible. Although the direct use of relationship matrices is a simple approach, the expansion of pedigree information utilization in GS across various machine learning methods is expected. Additionally, we proposed a new machine learning method, SAE, which explicitly incorporates interactions between variables (markers and/or relationships) into the model and confirmed its effectiveness. The proposed method achieved the highest accuracy for predicting some traits. SAE allows explicit modeling of interactions between markers and/or relationships, which may be suitable for sugarcane with complex genetic systems. Further evaluation is needed to determine if SAE surpasses additive GBLUP in other crops with simpler genetics.

This study did not incorporate the polyploidy of sugarcane by genotyping allele dosage. The potential and problems of “pseudo‐diploid modeling,” in which a predictive model is built for a polyploid organism as a diploid, have been discussed in the application of GS for sugarcane (Yadav et al., 2021). Statistical genetic methods designed specifically for polyploid crops have been proposed (de Bem Oliveira et al., 2019; Endelman et al., 2018), including the use of a marker system that can identify the dosage of each allele. A recent study (Batista et al., 2022) has applied these models, which account for polyploidy inheritance and marker systems capable of identifying doses, to sugarcane. This study underscores the importance of these models in capturing the complexity of polyploidy inheritance on certain traits. However, the accuracy of such approaches is contingent upon the validity of the model for a given trait and the precision of dose discrimination, emphasizing the necessity for further validation in future research endeavors. It is noteworthy that the machine learning methods proposed in this study can also be applied to marker genotypes considering dosage or genomic relationship matrices considering dosage. It is expected that such new genotyping and modeling techniques will contribute to improving the efficiency of sugarcane breeding in the future.

## AUTHOR CONTRIBUTIONS

**Minoru Inamori**: Conceptualization; data curation; formal analysis; methodology; validation; writing—original draft. **Tatsuro Kimura**: Conceptualization; data curation; funding acquisition; project administration; writing—original draft; writing—review and editing. **Masaaki Mori**: Conceptualization; data curation; writing—review and editing. **Yusuke Tarumoto**: Data curation; resources; writing—review and editing. **Taiichiro Hattori**: Data curation; resources; writing—review and editing. **Michiko Hayano**: Data curation; resources; writing—review and editing. **Makoto Umeda**: Data curation; resources; writing—original draft; writing—review and editing. **Hiroyoshi Iwata**: Conceptualization; data curation; investigation; methodology; project administration; supervision; visualization; writing—original draft.

## CONFLICT OF INTEREST STATEMENT

The authors declare no conflicts of interest.

## Supporting information

Supplemental Figure 1. Correlations among principal components based on the genomic and principal pedigree relationship matrices of 297 genotypes.

Supplemental Figure 2. Comparison of prediction accuracy between leave‐one‐family‐out cross‐validation and leave‐one‐family‐out cross‐validation with one randomly retained full sibling clone in the training data. The comparison was conducted on a subset of 10 families each containing 10 or more clones. To address randomness in the selection of one full sibling clone, the latter cross‐validation was repeated 100 times. For each repetition, accuracy was compared between the two cross‐validation schemes, and the difference between their accuracies was plotted as a bar graph. Results indicated that, across various traits, methods, and models, the accuracy of the latter was generally higher, suggesting that retaining one full sibling clone in the training data enhances accuracy. However, it was observed that in the case of SAE, retaining one full sibling clone in the training data led to a decrease in prediction accuracy.

## Data Availability

Estimated genotypic values, marker genotype scores, a genomic relationship matrix, a pedigree relationship matrix, and family IDs are accessible at https://doi.org/10.5061/dryad.0rxwdbs8p. The Python library for the newly developed SAE method is accessible at https://github.com/m‐inamori/SAEnsemble.
